# A Wind-Driven Rotating Micro-Hybrid Nanogenerator for Powering Environmental Monitoring Devices

**DOI:** 10.3390/mi13122053

**Published:** 2022-11-23

**Authors:** Yongqiang Zhu, Yu Zhao, Lijun Hou, Pingxia Zhang

**Affiliations:** School of Mechanical and Automotive Engineering, Qingdao University of Technology, Qingdao 266520, China

**Keywords:** hybrid nanogenerator, flexible contacts, rotational, wide-band wind speed, environmental monitoring

## Abstract

In recent years, environmental problems caused by natural disasters due to global warming have seriously affected human production and life. Fortunately, with the rapid rise of the Internet of Things (IoT) technology and the decreasing power consumption of microelectronic devices, it is possible to set up a multi-node environmental monitoring system. However, regular replacement of conventional chemical batteries for the huge number of microelectronic devices still faces great challenges, especially in remote areas. In this study, we developed a rotating hybrid nanogenerator for wind energy harvesting. Using the output characteristics of triboelectric nanogenerator (TENG) with low frequency and high voltage and electromagnetic generator (EMG) with high frequency and high current, we are able to effectively broaden the output voltage range while shortening the capacitor voltage rising time, thus obtaining energy harvesting at wide frequency wind speed. The TENG adopts the flexible contact method of arch-shaped film to solve the problem of insufficient flexible contact and the short service life of the rotating triboelectric generator. After 80,000 cycles of TENG operation, the maximum output voltage drops by 7.9%, which can maintain a good and stable output. Through experimental tests, the maximum output power of this triboelectric nanogenerator is 0.55 mW at 400 rpm (wind speed of about 8.3 m/s) and TENG part at an external load of 5 MΩ. The maximum output power of the EMG part is 15.5 mW at an external load of 360 Ω. The hybrid nanogenerator can continuously supply power to the anemometer after running for 9 s and 35 s under the simulated wind speed of 8.3 m/s and natural wind speed of 5.6 m/s, respectively. It provides a reference value for solving the power supply problem of low-power environmental monitoring equipment.

## 1. Introduction

With the rapid development of IoT technology, the demand for various sensors for environmental monitoring has increased dramatically in number. As the lifeblood of a sensing system, a reliable energy supply is a key factor for interactive communication. However, most of the current sensing networks rely mainly on batteries for their energy supply. On the one hand, the limited lifetime of batteries causes subsequent replacement and charging problems, resulting in intermittent data transmission. On the other hand, chemical batteries are difficult to adapt to various high and low temperatures and other complex environments and cause certain environmental pollution problems [1]. Therefore, the problem of power supply for sensing devices has become a major problem that hinders the further development of IoT systems, and electronic devices are in urgent need of more suitable power supply solutions. The use of obtaining environmental energy as a self-powering method for sensing nodes and electronic devices provides a new idea to solve the drawbacks of traditional power supply methods [2,3].

Wind energy has significant advantages in terms of wide distribution, resourcefulness, sustainability, and non-pollution [4]. The traditional method of converting wind energy into electricity is based on the principle of electromagnetic induction and turbine structure. The turbines of this wind turbine are complex and built with bulky magnet components. They are expensive, require high installation heights, and work with high start-up wind speeds. Based on these constraints, these types of wind turbines have not been fully utilized in distributed micropower sources and low-speed wind resources [5,6]. There is an urgent need for new methods to achieve efficient acquisition and in situ utilization of wind energy.

TENG is electrically generated by coupling triboeletrifiction [7,8] and electrostatic induction. It is a promising technology for converting ambient mechanical energy into electrical energy. It has been used to obtain energy from a variety of sources. It has the unique advantages of high power density, high efficiency, and low fabrication cost [9,10,11]. Due to its unique mechanism, TENG has higher performance than EMG in low-frequency environments, meaning that it may be the best application for low-frequency energy harvesting [12].

TENGs have high output voltages, but output currents are only in the μA level [13,14]. In contrast, EMGs have output currents up to the mA level [15,16,17,18]. However, electrical devices used for low power consumption use a DC power supply, the TENG and EMG generate AC power that needs to be converted to DC power through a rectifier bridge circuit. The existing rectifier bridge circuit will have a voltage drop of about 0.45 V, which will be very detrimental to the low voltage generated by EMG at low speed, and will have little effect on the high voltage output of TENG. At low-frequency movement, TENG is better than EMG for supplying energy to ultra-low power consumption appliances, but when the speed continues to rise, the advantages of the EMG high current will be revealed. The complementary outputs of TENG and EMG can be mixed and maximized to harvest green energy over a wide range of wind speeds. Of course, TENGs not only have the function of powering low-power electronic devices but also their applications in self-powered rotational speed detection, fluid rolling robots with voltage-driven oscillating liquids, and active sorting of droplets by using an electro-conjugate fluid micropump will become a hot topic of research. Hence, it remains a challenge to optimize the design and construction of hybrid structures for their different usage scenarios and movement pattern characteristics. The main hybrid nanogenerators currently available for wind energy harvesting are thin-film chattering [19,20], rotary-contact separation [21,22,23], and rotating structures [24,25,26,27,28,29,30,31].

**Figure 1:**
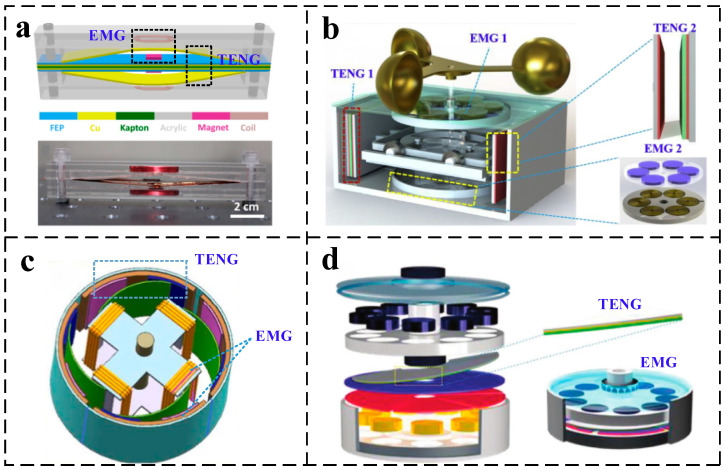
Various structures of hybrid nanogenerators (**a**) Film chattering type [19] (**b**) Rotary-contact separation type [21], and (**c**,**d**) Rotational [27,28].

Wang [19] et al. designed a thin-film chattering hybrid nanogenerator with the upper and lower electrode films as the basic mechanism, as shown in Figure 1a. The wind energy is first converted into vibration energy of the upper and lower electrodes by vibrating the film, and then the electrical energy is generated by the relative motion of the film during vibration. However, the irregular chattering motion causes the output performance of the chattering type to be difficult to keep stable. And this open structure causes water droplets and dust to adhere to the electrode surface, requiring long-term maintenance work. Therefore, this lower output performance with the open structure leads to the thin-film chattering type hybrid nano-generation cannot meet the energy supply demand of monitoring devices in complex environments. Fan [21] et al. designed a rotating-contact separation hybrid nano-generator that converts the rotational motion into linear reciprocating motion, as shown in Figure 1b. The magnet in the EMG maintains relative rotational motion with the coil, and the TENG with two electrodes of mutual contact separation generates an output voltage. This structure has good output performance at low wind speeds. However, due to the existence of motion mode switching, electrostatic adsorption, and collision effect during contact separation, which will consume a lot of mechanical energy to reduce the motion frequency. In the ultra-high wind speed condition of 16 m/s, the generator speed is 250 rpm, which is not conducive to the output of EMG. In conventional rotating structures, TENGs mostly use paddle-type flexible contact or disc-type rigid contact. Wang [27] et al. designed paddle-type TENG structure with flexible contact is prone to insufficient contact problems such as point contact and line contact between triboelectric electrodes in lower motion frequencies, as shown in Figure 1c. The maximum output voltage of the TENG is 35 V at 200 rpm, which is not conducive to the collection and utilization of low-speed wind by the hybrid nanogenerator. Zhang [28] et al. used a disc-type flat contact structure in a rotating TENG, as shown in Figure 1d. An open-circuit voltage of 80 V can be output at 300–900 rpm, but the durability of the TENG was not verified, and the poor durability of the TENG is a major hindrance to the application of this structure. Xu [29] et al. used an open windmill shape to combine the TENG with the EMG, which is poorly adaptable in outdoor environments. Zhang [30] et al. designed a wind energy generator applied to a high-speed train system, and the performance of the TENG was maintained at 89.04% of the original after 50,000 cycles. Wang [31] et al. combined the rotating type with the gravitational potential energy of a heavy object, and the complex gearing made the mechanical energy loss huge.

Based on the above research results, the motion characteristics of the rotating mechanism are combined. We have studied a fully enclosed, wide operating frequency and durable rotating hybrid nanogenerator. It consists of an arch-shaped structure of a TENG part and a rotating EMG part. Compared with the thin-film chattering hybrid nanogenerator, it has a fully enclosed structure, which reduces the influence of environmental factors on the power generation performance. It solves the problem of large mechanical energy loss of the rotating-contact separated hybrid nanogenerator, which is unfavorable to wind energy harvesting by EMG under high-speed wind. For the conventional rotating hybrid nanogenerator, due to the structure of the arch-shaped film in the TENG, which solves the problem of insufficient contact in the paddle-type flexible contact. Compared with disc-type rigid contact, flexible contact reduces frictional resistance between triboelectric materials and has good durability. In addition, the disk-based EMG also does the rotational motion synchronously to improve the hybrid output performance of the wind energy generator. In this paper, the open-circuit voltage and output power of the optimized hybrid nanogenerator are tested. And the durability of this hybrid nanogenerator is analyzed by persistence test. On this basis, the applicability of the device is proved by charging the capacitor and lighting the light-emitting diode (LED). Finally, we conducted experiments under simulated and natural wind conditions and confirmed that the device reached the capability of powering a commercial anemometer after running for 9 s at a wind speed of 8.3 m/s and 35 s at a wind speed of 5.6 m/s, respectively. Therefore, this hybrid nanogenerator can provide some reference value for solving the power supply problem of environmental monitoring equipment.

## 2. Fabrication and Working Principles

### 2.1. Fabrication

**Figure 2:**
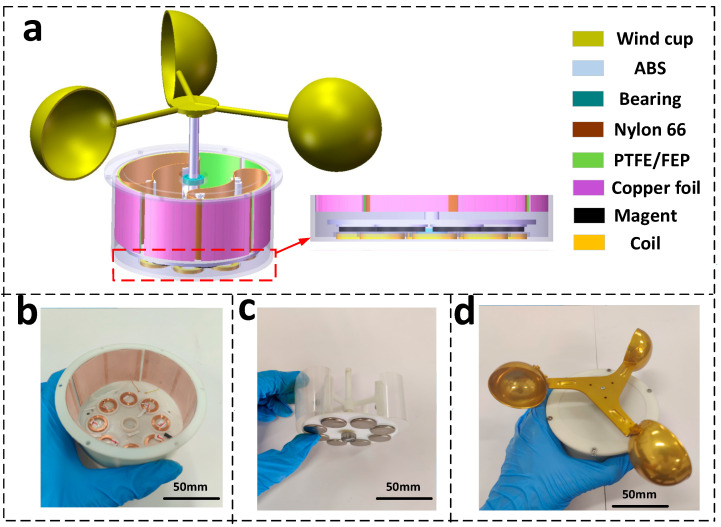
Device schematic of hybrid nanogenerator (**a**) Schematic diagram of the hybrid nanogenerator, (**b**) Stator, (**c**) Rotors, and (**d**) Overall assembly diagram.

The device schematic of the hybrid nanogenerator is shown in Figure 2. The generator mainly consists of stator, rotor, and wind cups. The stator is a cylindrical shell with an outer diameter of 100 mm, an inner diameter of 96 mm, and a height of 55 mm. Copper foil of size 70 × 40 × 0.1 mm is pasted on the inner wall of the stator. The copper foil is covered with a negative polymer film to form a conductive layer and a negative triboelectric layer of the TENG. The conductive layer and the negative polymer film form an electrode, and the total number of electrodes in the device is four. Eight series-connected magnetic induction coils are placed in a circular arrangement in a groove at the bottom of the stator. The rotor consists of a shaft, a central cross-shaped support structure, and a disk at the bottom. The cross-shaped bracket is fitted with two arch-shaped nylon 66 films as positive triboelectric electrodes. To create good contact between the positive and negative polymer films, the outer diameter of the arch formed by nylon 66 film is 98 mm, and the disk at the bottom of the rotor is fitted with the same number of cylindrical permanent magnets as the magnetic induction coil, with N-S poles placed alternately adjacent to the permanent magnets. The magnets are positioned perpendicular to the center of the coil so that the coil can be cut off by the edge of the strongest magnetic field. The bearings are mounted on the rotor shaft end, and the rotor is driven by the wind cup for rotational motion.

In this study, the stator and rotor are manufactured by 3D printing technology, and the printing material is ABS resin. The selected triboelectric materials are nylon 66, Polytetrafluoroethylene (PTFE), and Fluorinated ethylene propylene (FEP). Nylon 66 is a positive triboelectric material with a thickness of 0.3 mm. PTFE and FEP are negative triboelectric materials. Nylon 66 and two negative polymer materials belong to the opposite spectra of the triboelectric series [32,33]. NdFeB-N35 was chosen as the magnet material because of its high magnetic field strength. The specific dimensions are 20 mm in diameter and 2 mm in thickness. The magnetic induction coils have an outer diameter size of 18.5 mm, an inner diameter of 12.5 mm, a height of 2.3 mm, a wire diameter of 0.1 mm, and 480 turns per coil. The bearings are low-resistance deep groove ball bearings (XUDZ, Japan) with an outer diameter of 13 mm and an inner diameter of 6 mm. The specific dimensions and values of each component are shown in Table 1.

In this device, the magnet and coil form the electromagnetic generation section, and the flexible nylon 66 film and PTFE/FEP film form the sliding-independent layer triboelectric nanogeneration section.

### 2.2. Working Principle

**Figure 3:**
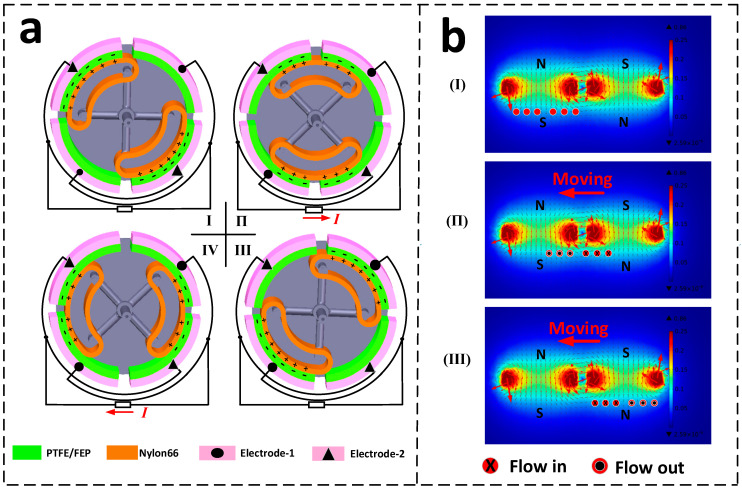
The principle of TENG and EMG (**a**) The principle of TENG: (I–IV) charge transfer forming reverse current and (**b**) The principle of EMG: (I–III) diagram of the formation of current and the change of current direction.

The working principle of the hybrid nanogenerator for wind energy harvesting is shown in Figure 3. The working principle is elaborated from two aspects of the triboelectric-based electrical effect and the electromagnetic-based effect, or power generation principle. Regarding wind energy harvesting by triboelectric electricity generation, the power generation process is elaborated through a basic unit as shown in Figure 3a. In the original state (stage I), when the nylon 66 film is in contact with the negative film on electrode-1. Due to their different electron affinities, the charge transfer at the contact interface will cause PTFE or FEP to have negative charges and nylon 66 film to have positive charges. These triboelectric charges will be maintained on the material surface for a longer period of time. In the corresponding position, the positive triboelectric charges fully compensate for the negative triboelectric charges. When the rotor starts to rotate, relative sliding occurs between the triboelectric materials, and the triboelectric charges generated in the sliding area are not compensated. As the rotor rotates to stage П, electric charges in electrode-2 flow into electrode-1 through an external circuit. As the rotor rotates to stage III, the nylon66 film is in complete contact with the negative film on electrode-2 and the charges are in a new equilibrium state, so there is no charge flow in the circuit. Further rotating to stage IV, the charges of electrode-1 are transferred to electrode-2, thus creating a reverse current between the two electrodes. Finally, it rotates again to the state shown in stage-Ⅰ and a new operating cycle begins.

Wind energy harvesting is based on the electromagnetic effect, according to Faraday’s principle of electromagnetic induction. We have simulated the magnetic flux distribution of adjacent cylindrical permanent magnets (magnets arranged in alternating polarity) using COMSOL software, and the flux distribution is shown in Figure 3b. During the simulation, the cylindrical housing is used as the outer boundary, and the space is set to the air domain. The permanent magnet size is the same as the real size, the defined material is NdFeB-N35, and the residual magnetic flux intensity is 1.28 T. A free triangular mesh of standard cell size is used to divide the region. The simulation finds that the magnetic field is strongest at the edges of the permanent magnets. And the edges of two adjacent magnets form a closed magnetic circuit, which further increases the magnetic field strength of the magnet edges. Constrained by the internal space limitation, we arranged the magnets and coils in parallel. In stage Ⅰ, the magnets are aligned with the coil, there is no current in the coil, and the magnetic flux through the coil is small. When the magnet on the rotor rotates from stage Ⅰ to stage П, the magnetic flux through the coil will gradually increase, resulting in a positive induced current in the coil. While the magnet rotates from stage П to stage III, the magnetic flux through the coil decreases, and a negative induced current is generated in the coil.

## 3. Electrical Measurement and Method

**Figure 4:**
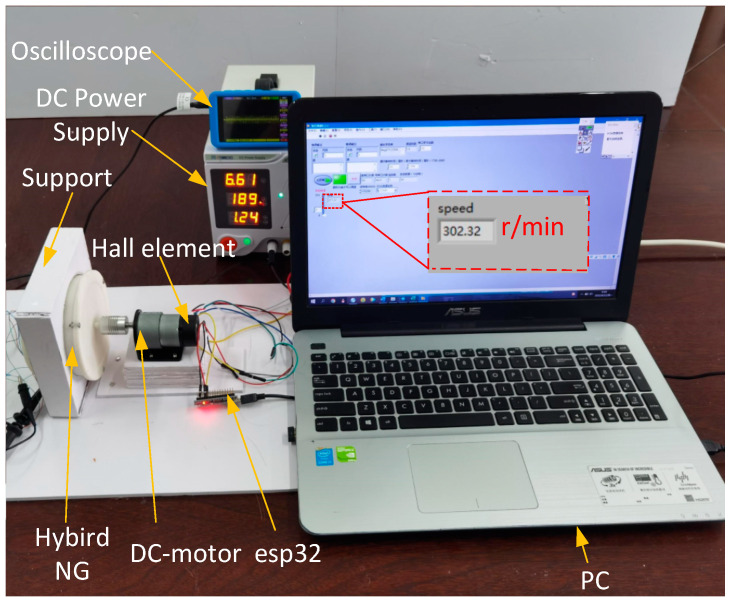
Experimental setup diagram.

In electrical measurements of hybrid nanogenerators. A DC power supply is used to drive the hybrid nanogenerator via a DC motor (CHR-GM37-520, Chihai electric machinery co., Ltd., Shenzhen, China) for rotational motion at different operating speeds. The rotational speed is collected by the hall element and transmitted back to the Labview interface in the PC through the microcontroller esp32 for displaying the rotational speed. An oscilloscope (DS100 Mini Guangzhou, Xingyi Electronic Technology Co., Ltd., Guangzhou, China) was used to evaluate the open circuit voltage and the experimental equipment is shown in Figure 4. The type and thickness of the negative triboelectric materials in the TENG are compared and experimented with, and finally, the optimal material type and thickness are prepared for the TENG. The output of the hybrid nanogenerator is tested at different rotational speeds and different external loads, and the output voltage of the generator is recorded.

The following experimental data were collected from the hybrid nanogenerator after one minute of stable operation.

## 4. Results and Discussion

### 4.1. Output Characterization

**Figure 5:**
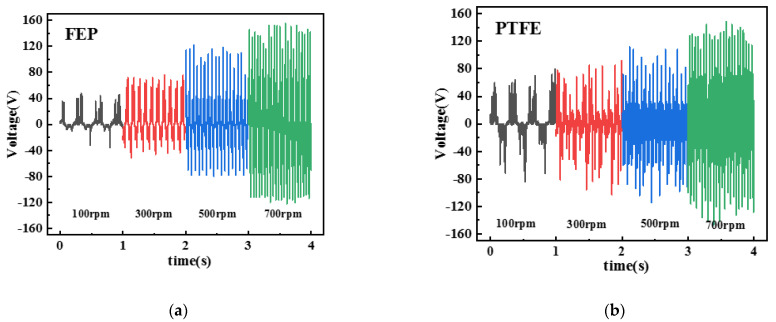
Power generation performance with different materials (**a**) FEP film and (**b**) PTFE film.

In order to obtain better output performance, this study first conducted a comparative experiment on the types of triboelectric materials. PTFE and FEP with 0.1 mm thickness were selected as negative triboelectric materials for power generation performance testing, and the results are shown in Figure 5.

The TENG was tested in the speed range of 100–700 rpm in steps of 200 rpm for different materials. It was demonstrated that the open-circuit voltage of PTFE is significantly higher than that of FEP at 100–300 rpm. At 500–700 rpm, the open-circuit voltage using PTFE as triboelectric material is about the same as that using FEP as triboelectric material. Considering that the advantage of TENG is its high-power generation capacity at low wind speed, PTFE was chosen as the negative triboelectric material.

In the next experiments, we investigated the effect of different thicknesses of PTFE materials on the power generation efficiency of TENG. A constant rotational speed of 100 rpm was selected for the test, and three different thicknesses of PTFE, 0.05 mm, 0.1 mm, and 0.2 mm, were used to test the open circuit voltage of the TENG.

**Figure 6:**
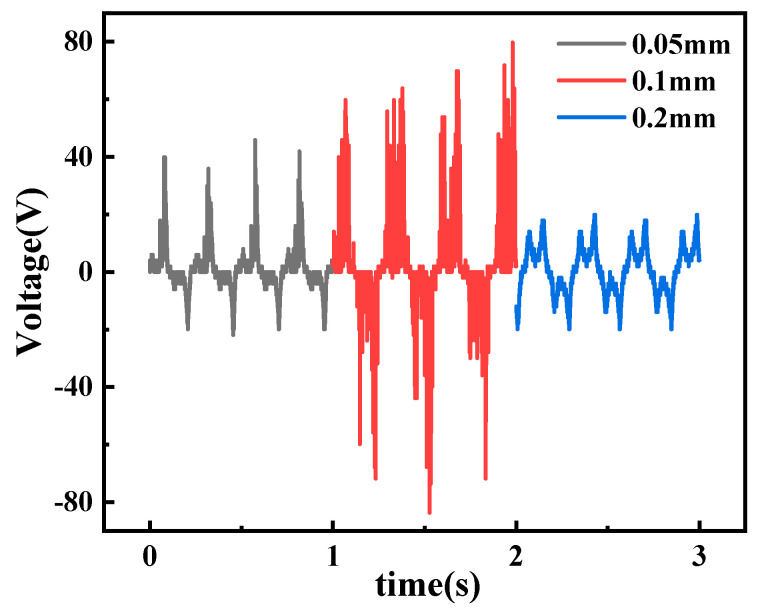
Electricity generation performance of PTFE materials at different thicknesses.

As shown in Figure 6, varying the thickness of the PTFE had a significant effect on the power generation performance of the TENG, with all other conditions remaining the same. The TENG using 0.1 mm thickness of PTFE with nylon 66 produced the best power generation capability with a peak voltage of 79.9 V. The peak voltage generated by the TENG using 0.05 mm thickness PTFE was 53.8 V and the peak voltage generated by the TENG using 0.2 mm thickness PTFE was 21.9 V. The reason is that PTFE produces more folds at 0.05 mm thickness, which causes the contact area between positive and negative friction materials to decrease, resulting in poor TENG power generation performance. As the thickness increases, the flatness of the material surface improves, the contact area between the positive and negative triboelectric materials increases, and the performance of TENG power generation is improved. When the thickness exceeds 0.1 mm, the material is insulating, resulting in a large amount of charge generated by triboelectric gathering on the triboelectric surface, which cannot cross the film into the copper electrode and form a charge flow with the external circuit, causing a decrease in the amount of transferred charge and further leading to a decrease in voltage. Through the above-mentioned comparative experiments, PTFE with 0.1 mm thickness was selected as the negative triboelectric material for subsequent experiments in this study.

**Figure 7:**
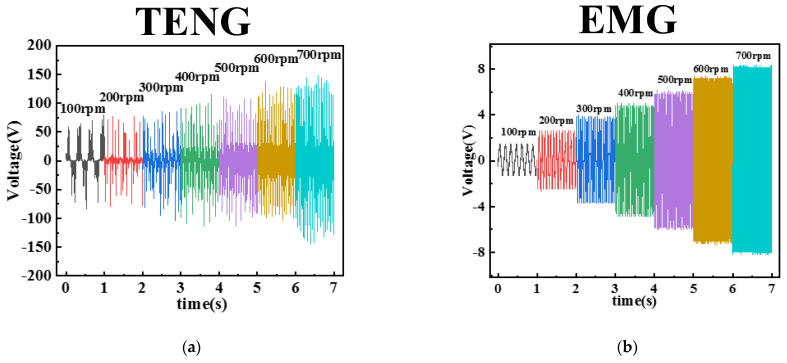

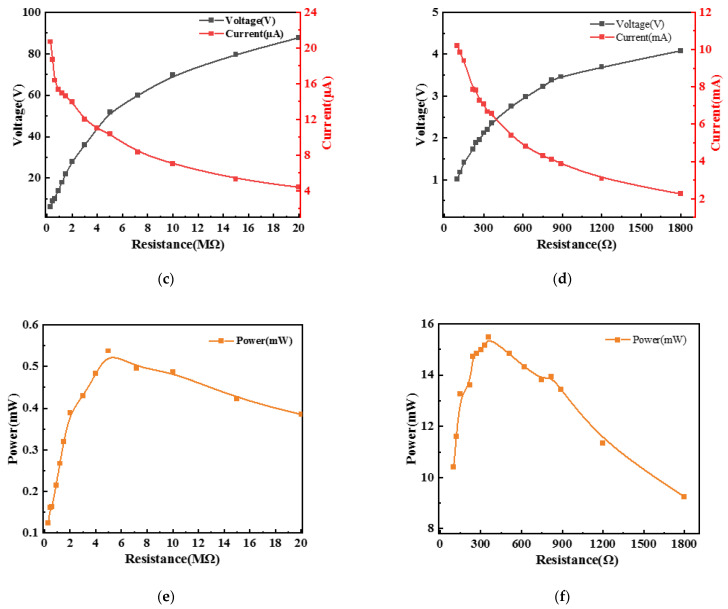
Electrical output performance of the TENG and EMG of the hybrid nanogenerator (**a**,**b**) Open circuit voltage versus speed for TENG and EMG, (**c**,**d**) Variation curves of voltage, current and external resistance of TENG and EMG at 400 rpm, and (**e**,**f**) Output power of TENG and EMG versus external resistance.

In order to characterize the hybrid nanogenerator for wind energy harvesting, its output performance was investigated. Figure 7a illustrates the dependence of the open-circuit voltage of the TENG on the rotational speed in the speed range of 100–700 rpm. Referring to the literature [34,35], the open-circuit voltage amplitude for a rotating TENG is calculated as

(1) $$V^{TENG}=\frac{\sigma S}{C}$$

where *σ* is the surface charge density of the polymer film, *S* is the contact area of the two triboelectric materials, and *C* is the capacitance of the two adjacent electrodes. The output voltage magnitude is highly dependent on the maximum value of the transmitted charge, which is proportional to the relative contact area *S* between the triboelectric materials and the surface charge density *σ*.

In the rotational speed interval below 500 rpm. The surface charge of the frictional triboelectric materials increases with the speed, the surface charge density *σ* increases, and the maximum open-circuit voltage of the TENG slowly increases from 79.7 V to 126 V. In the speed interval above 500 rpm. As the rotational speed continues to increase, the maximum open-circuit voltage of TENG increases rapidly from 126 V to 159 V. The reason is that at low speed, the arch-shaped nylon film can produce good contact with the PTFE film on the inner wall, and the relative contact area *S* does not change much. In the case of high rotational speed, the nylon film is deformed due to centrifugal force. The relative contact area *S* between the triboelectric materials becomes larger, which leads to a rapid increase in voltage. Figure 7c,e show the output voltage and current of the TENG at 400 rpm speed. The output power of the TENG is further investigated using a resistor as an external load for the conversion of mechanical motion to electrical energy. As the external load resistance increases from 290 kΩ to 20 MΩ, the current amplitude decreases from 20.7 μA to 4.4 μA. At a load resistance of 5 MΩ, the maximum output power of the TENG is 0.55 mW.

In addition, we have also characterized the output performance of the EMG section systematically, as shown in Figure 7b. With the help of Faraday’s law [36], the open-circuit voltage of the EMG can be calculated as

(2) $$V^{EMG}=-nN\frac{d\emptyset_{B}}{dt}$$

where *n* is the number of coils, *N* is the number of turns per coil and $\emptyset_{B}$ is the total magnetic flux in each coil. Obviously, the rate of change of the magnetic flux is related to the speed.

From Equation (2), the open-circuit voltage of the EMG is proportional to the rotational speed. In this experiment, *n* is 8 and *N* is 480. The maximum open-circuit voltage is from 1.47 V to 8.57 V in the speed interval of 100–700 rpm. The linear relationship between the open-circuit voltage and the rate of change of magnetic flux $d\emptyset_{B}/dt$ in the above equation is satisfied. Similarly, we use external resistors to evaluate the output performance of the EMG, as shown in Figure 7d,f. At 400 rpm, as the external load resistance increases from 100 Ω to 1800 Ω, the EMG output voltage increases from 1.02 V to 4.08 V and the output current amplitude decreases from 10.2 mA to 2.3 mA. at a load resistance of 360Ω, the EMG maximum instantaneous power is up to 15.5 mW.

**Figure 8:**
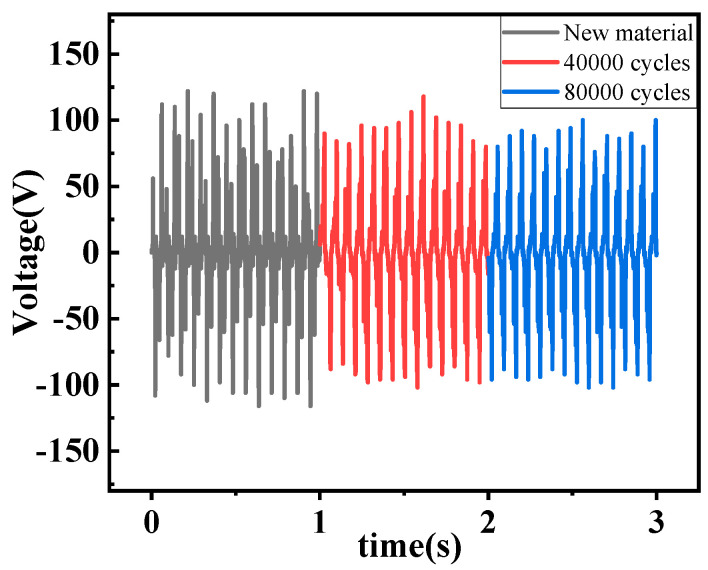
Durability test.

The decrease in power generation capacity after continuous operation is one of the main problems of the rotating TENG. We sampled and collected the output voltage of TENG after the new material, 40,000 cycles, and 80,000 cycles, and the results are shown in Figure 8. Black represents the maximum output voltage of TENG with new material is 126 V. Red represents the output voltage of TENG after 40,000 cycles of testing. The maximum voltage that can be generated is 120 V. Blue represents that the maximum output voltage of TENG after 80,000 cycles of testing is 116 V. The results found that the triboelectric material in the proposed arch flexible contact method of TENG has a good service life, and its maximum output voltage decreases by 7.9% after 80,000 cycles.

### 4.2. Demonstration

**Figure 9:**
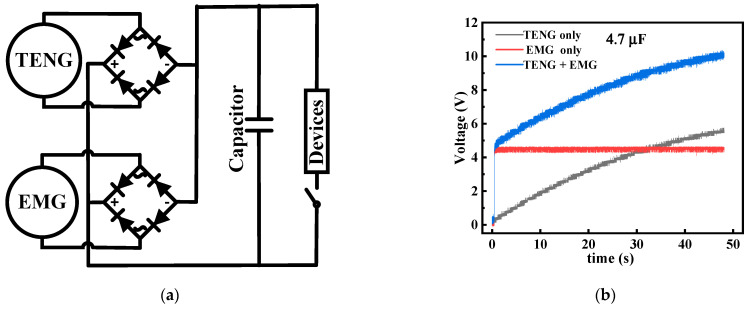

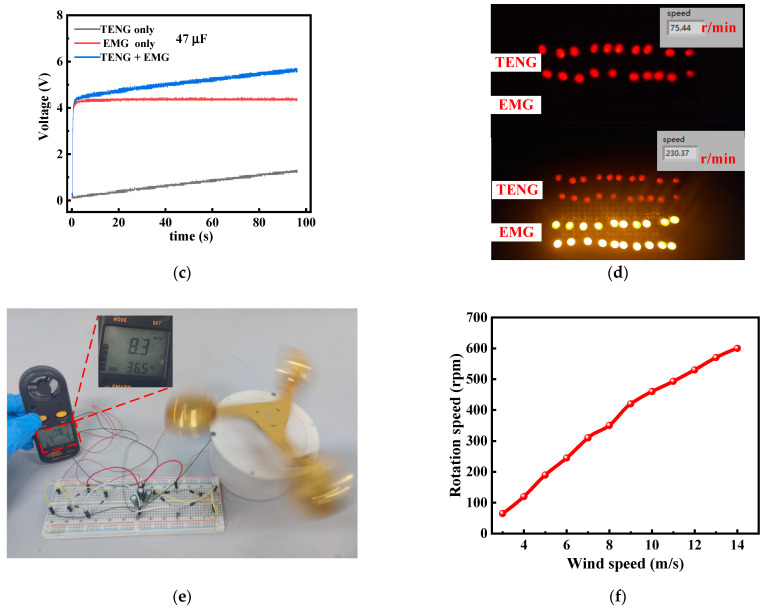
Demonstration of a hybrid nanogenerator as a practical power source (**a**) Capacitor charging and load supply circuit diagram, (**b**) Charging curve of 4.7 μF capacitor, (**c**) Charging curve of 47 μF capacitor, (**d**) Picture of 20 leds lit by TENG or EMG and the corresponding RPM, (**e**) Energy supply for small devices, and (**f**) Relationship between rotational speed and wind speed.

In general, the energy conversion device should have good stability and consistency. In the charging test, the hybrid nanogenerator was first used to charge capacitors of different capacities. To obtain the DC output from TENG and EMG, the conversion of AC signals has been achieved with two bridge rectification circuits. Figure 9a shows the schematic diagram of the rectifier circuit. When the speed is 400 rpm, the hybrid nanogenerator charges the capacitors of 4.7 μF and 47 μF capacity, and the charging curves are shown in Figure 9b,c respectively. The gray curve represents the TENG part, the red curve represents the EMG part, and the blue curve represents the combined charging of TENG and EMG. Since the EMG has a high output current and low output voltage. When the EMG charges the capacitor, the voltage of the capacitor quickly rises to about 4.3 V output voltage, and then the voltage basically remains the same. The output voltage of the EMG limits the maximum voltage of the capacitor, resulting in a large energy loss. In addition, the TENG has the electrical characteristics of high output voltage and low output current. After the output voltage of TENG in the hybrid nanogenerator reaches the limit of the EMG output voltage, it is still possible to continue to increase the voltage of the capacitors. In addition, due to the electrical characteristics of TENG with high output voltage and low output current, the voltage of the TENG part of the hybrid nanogenerator can continue to increase the voltage of the capacitor even after the voltage of the EMG part reaches the limit of the output voltage. Therefore, combining TENG with EMG can provide fast charging speed and charging voltage. In addition, when the hybrid nanogenerator charges capacitors of different capacities, the charging voltage is mainly provided by the TENG after the charging voltage reaches the limit voltage of the EMG, and the charging speed decreases with the increase of the capacitor capacity. It indicates that the main charging property changes from EMG to TENG at the later stage of charging. The results show that the combination of TENG and EMG has a significant improvement in energy harvesting and conversion compared to the energy harvesting of each single part. We found that the hybrid nanogenerator can easily output a much wider voltage range by charging capacitors of different capacities experimentally. The power supply needs of different devices in the external circuit are met without any additional circuit changes.

In addition to charging capacitors, this hybrid nanogenerator can also supply power to small electronic devices. Taking lighting LEDs as an example, the TENG is suitable for the series powering of LEDs due to its high voltage characteristics. The EMG has high current characteristics and is suitable for the parallel powering of LEDs. The LEDs are all of the same type, with red and yellow colors at voltages of 1.8 V to 2.0 V operating voltage, as shown in Figure 9d. At 75 rpm, TENG was the first to light up 20 red LEDs. Hence, the TENG is already capable of driving small electronic devices at very low operating frequencies. At 230 rpm, the EMG lights up 20 yellow LEDs. The yellow LEDs driven by the EMG have a stronger brightness when lit, indicating that the EMG has a stronger power generation capability at higher speeds or higher operating frequencies. Therefore, the TENG part and the EMG part have good complementarity in providing energy in a wider frequency range of rotational speed. We used this hybrid nanogenerator to power a commercial anemometer to detect the wind speed, as shown in Figure 9e. A wind speed of 8.3 m/s, after operating for 9 s, successfully powers the anemometer. The relationship between wind speed and generator speed was measured as Figure 9f, and the wind speed was basically linear with the speed.

**Figure 10:**
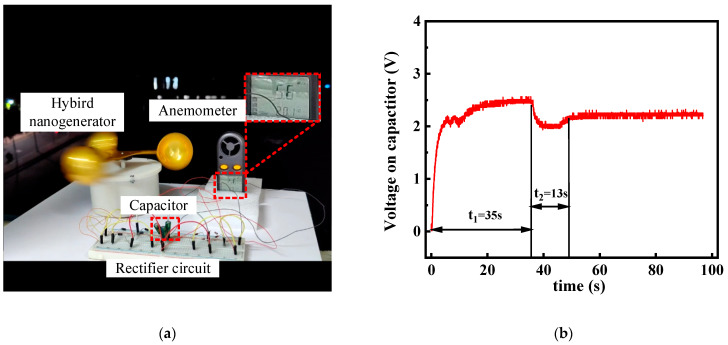
Demonstration in natural wind environment (**a**) Natural wind energy harvesting and anemometer power supply, as well as (**b**) Voltage curve on the capacitor.

Finally, to show the application potential in a real environment, we conducted experiments on wind energy harvesting in a natural wind environment. As shown in Figure 10a, the output of the hybrid nanogenerator is converted from AC voltage to DC voltage by two different rectification circuits, which together charge the capacitor. The positive and negative terminals of the capacitor are connected in parallel with the positive and negative terminals of the anemometer to realize the power supply to the anemometer. In addition, we recorded the voltage variation curves at the terminals of the capacitor as shown in Figure 10b. Running continuously for 35 s at a natural wind speed of about 5.6 m/s, the voltage across the capacitor rises sharply and then slowly, finally reaching the minimum starting voltage of 2.5 V for the anemometer, which proves the high charging efficiency of the EMG at the same frequency and the continuous boosting feature of the TENG. The hybrid output shortens the voltage rise time and widens the voltage output range. The voltage across the capacitor drops to 2 V due to the higher power required for anemometer start-up. After 13 s, the voltage across the capacitor is smoothly maintained at about 2.2 V, achieving a dynamic balance between charging and discharging of the capacitor.

In summary, the results show that the hybrid nanogenerator has good potential for application in solving the power supply of low-power environmental detection devices, especially in remote mountainous areas, deserts, and oceans.

## 5. Conclusions

In this paper, a rotating triboelectric-electromagnetic hybrid power generation device for wind energy harvesting and self-powered environmental monitoring is proposed. In this hybrid nanogenerator, the design of the arch-shaped structure in the TENG part results in a good contact area between the triboelectric materials. Meanwhile, the flexible polymer film reduces the frictional resistance and solves the problem of severe triboelectric loss of TENG. Comparative experimental results show that the TENG output prepared from PTFE films of 0.1 mm thickness is higher than that of other thicknesses with other kinds of polymer films. The maximum output power that the TENG and EMG can produce is 0.55 mW and 15.5 mW at an external load of 5 MΩ and 360 Ω respectively. The maximum voltage output of the triboelectric nanogenerator decreases to 7.9% after 80,000 cycles of continuous operation. In experiments on charging capacitors, the hybrid nanogenerator has a wider voltage range and shorter capacitor voltage rise time compared to individual generator sets.

To prove the feasibility of its practical application, we conducted an LED lighting test. At 230 rpm, both TENG and EMG can light up 20 LEDs. At 75 rpm, only the TENG was able to light 20 LEDs, proving that the TENG can collect energy more efficiently than the EMG in a low-frequency speed environment. In addition, the hybrid nanogenerator was successfully realized to power a commercial anemometer after operating at a simulated wind speed of 8.3 m/s for 9 s time and at a natural wind speed of 5.6 m/s for 35 s, respectively. In summary, this study provides a new solution to the power supply problem of self-powered environmental equipment monitoring based on wind energy harvesting.

## Figures and Tables

**Table 1:** Dimensions and values of each component.

Structural Parts	Parameters	Value
Stator	Outer diameter (mm)	100
	Inner diameter (mm)	96
	Height (mm)	55
FEP/ Copper coil	Length (mm)	70
	Width (mm)	40
	Thickness (mm)	0.1
PTFE	Length (mm)	70
	Width (mm)	40
	Thickness (mm)	0.05/0.1/0.2
Nylon 66	Width (mm)	40
	Thickness (mm)	0.3
	Arch outer diameter(mm)	98
Wind cup	Diameter (mm)	200
Magnet (NdFeB-N35)	Diameter (mm)	20
	Thickness (mm)	2
	Residual magnetic strength (T)	1.28
Coil	Outer diameter (mm)	18.5
	Inner diameter (mm)	12.5
	Number of turns	480
Distance between magnet and coil	d(mm)	0.5
Bearing	Outer diameter (mm)	13
	Inner diameter (mm)	6
